# Supplementary material for: Time-Dependent Efficacy of Checkpoint Inhibitor Nivolumab: Results from a Pilot Study in Patients with Metastatic Non-Small-Cell Lung Cancer
Source: Cancers (Basel). 2022 Feb 11;14(4):896. doi: 10.3390/cancers14040896 (PMC8870559; doi:10.3390/cancers14040896)
Supplement: Supplementary file 1 [file cancers-14-00896-s001.zip › cancers-1553278-supplementary.pdf]

cancers

MDPI

Supplementary data

Table S1: Main baseline characteristics of whole study population, and according to nivolumab (NIV) timing groups

Characteristics	All patients (N = 95)	Group 1[a] (n = 36)	Group 2 (n = 24)	Group 3 (n = 35)	p-value
**Actual NIV[b] timing**					
Median time, hh:min	12:54	11:29	12:57	13:53	
(range)	(9:27–17:14)	(9:27–16:46)	(09:45–16:52)	(10:10–17:14)	
(IQR)	(11:55–13:34)	(11:14–12:18)	(12:40–13:10)	(13:17–14:49)	
Median intra-patient CV[c]	10%	11%	11%	7%	
(range)	(2%–21%)	(2%–21%)	(8%–13%)	(9%–19%)	
**Age, years**					
Median (range)	66.9 (41.2–82.5)	67.2 (41.2–80.8)	65.8 (48.8–82.5)	69.2 (48.8–82.5)	0.272012
**Sex**					
Female	16 (16.8%)	4 (11.1%)	2 (8.3%)	10 (28.6%)	0.088
Male	79 (83.2%)	32 (88.9%)	22 (91.7%)	25 (71.4%)	
**Post-smoking COPD[d]**	74 (77.9%)	27 (75.0%)	20 (83.3%)	27 (77.1%)	0.741
**Histological type**					
Adenocarcinoma	55 (57.9%)	20 (55.6%)	12 (50.0%)	23 (65.7%)	
Squamous cell carcinoma	37 (38.9%)	14 (38.9%)	12 (50.0%)	11 (31.4%)	0.563
NSCLC unspecified	3 (3.2%)	2 (5.6%)	0 (0.0%)	1 (2.9%)	
**PD-L1**					
⩾ 1%	39 (41.1%)	19 (52.8%)	9 (37.5%)	11 (31.4%)	
< 1%	33 (34.7%)	9 (25.0%)	10 (41.7%)	14 (40.0%)	0.173
Not assessed	23 (24.2%)	8 (22.2%)	5 (20.8%)	10 (28.6%)	
**Primary resected**	15 (15.8%)	6 (16.7%)	5 (20.8%)	4 (11.4%)	0.637
**Prior adjuvant chemo.**	11 (11.6%)	6 (16.7%)	2 (8.3%)	3 (8.6%)	0.555
**Prior radiotherapy**	57 (60.0%)	19 (52.8%)	18 (75.0%)	20 (57.1%)	0.207
**N of prior chemo. lines**					
0	1 (1.1%)	1 (2.8%)	0 (0.0%)	0 (0.0%)	
1	72 (75.8%)	27 (75.0%)	16 (66.7%)	29 (82.9%)	0.345
2-5	22 (23.2%)	8 (22.2%)	8 (33.3%)	6 (17.1%)	
**Number of sites involved**					
Median (range)	4 (1–8)	4 (1–7)	4 (2–6)	3 (2–8)	0.985
**Site of metastases**				8 (22.9%)	

Brain	23 (24.2%)	7 (19.4%)	8 (33.3%)	16 (45.7%)	0.456
Pleura	39 (41.1%)	10 (27.8%)	13 (54.7%)	20 (57.1%)	0.098
Bone	49 (51.6%)	15 (41.7%)	14 (58.3%)	7 (20.0%)	0.318
Liver	24 (25.3%)	15 (41.7%)	2 (8.3%)	9 (25.7%)	0.010
Adrenal gland	19 (20.0%)	9 (25.0%)	1 (4.2%)	2 (5.7%)	0.065
Pericardium	7 (7.4%)	2 (5.6%)	3 (7.4%)		0.542
**WHO performance status**					
0	35 (36.8%)	17 (47.2%)	8 (33.3%)	10 (28.6%)	
1	56 (58.9%)	17 (47.2%)	16 (66.7%)	23 (65.7%)	0.347
2	4 (4.2%)	2 (5.6%)	0 (0.0%)	2 (5.7%)	

[a]Group 1, 67% of infusion before 12:54; Group 2, 33% of infusion before and 33% of infusion after 12:54; Group 3, 67% of infusion after 12:54; [b]Nivolumab; [c]Coefficient of variation; [d]Chronic obstructive pulmonary disease.
